# Retraction Note: The toxicity and therapeutic effects of single-and multi-wall carbon nanotubes on mice breast cancer

**DOI:** 10.1038/s41598-023-46596-w

**Published:** 2023-11-15

**Authors:** Arghavan Kavosi, Saeideh Hosseini Ghale Noei, Samaneh Madani, Solmaz Khalighfard, Saeed Khodayari, Hamid Khodayari, Malihe Mirzaei, Mohammad Reza Kalhori, Majid Yavarian, Ali Mohammad Alizadeh, Mojtaba Falahati

**Affiliations:** 1 Department of Cellular and Molecular Biology, Faculty of Advanced Sciences and Technology, Islamic Azad University, Pharmaceutical Science Branch, Tehran, Iran; 2 Department of Biology, Islamic Azad University, Science and Research Branch, Tehran, Iran; 3 Cancer Research Center, Tehran University of Medical Sciences, Tehran, Iran; 4 Breast Disease Research Center, Tehran University of Medical Sciences, Tehran, Iran; 5 Department of Biology, Islamic Azad University, Arsanjan Branch, Arsanjan, Iran; 6 Hematology Research Center, Shiraz University of Medical Sciences, Shiraz, Iran; 7 Department of Nanotechnology, Faculty of Advance Science and Technology, Pharmaceutical Sciences Branch, Islamic Azad University, Tehran, Iran

Retraction of: *Scientific Reports* 10.1038/s41598-018-26790-x, published online 30 May 2018

The Editors have retracted this Article. Several panels in Figure 10 are also published in Figure 1 of an Article that was simultaneously under consideration at a different journal by overlapping Authors [1]. Specifically:

- The 21 day Tumor panel appears to be identical to the 21 day control panel of Figure 1 in [1]
- The 29 day Tumor panel appears to be identical to the 29 day ATO panel of Figure 1 in [1]
- The 39 day Tumor panel appears to be identical to the 28 day Control panel of Figure 1 in [1]
- The 14 day CNTs panel appears to be identical to the 14 day OT panel of Figure 1 in [1]
- The 29 day CNTs panel appears to be identical to the 28 day OT panel of Figure 1 in [1]
- The 39 day CNTs panel appears to be identical tot the 42 day OT panel of Figure 1 in [1]

Further checks by the Publisher have found that the error bars in all Figures are ± 5%, not SD as stated in the Materials and Methods and Figure legends.

The Editors therefore no longer have confidence in the results and conclusions of this Article.

Ali Mohammad Alizadeh does not agree to this retraction. Mojtaba Falahati has not explicitly stated whether they agree to this retraction. The Publisher has not been able to obtain a current email address for Samaneh Madani. None of the other authors have responded to any correspondence from the Publisher about this retraction.
